# Qwen-2.5 Outperforms Other Large Language Models in the Chinese National Nursing Licensing Examination: Retrospective Cross-Sectional Comparative Study

**DOI:** 10.2196/63731

**Published:** 2025-01-10

**Authors:** Shiben Zhu, Wanqin Hu, Zhi Yang, Jiani Yan, Fang Zhang

**Affiliations:** 1 Department of Infectious Diseases, Nanfang Hospital Southern Medical University Guangzhou China; 2 State Key Laboratory of Organ Failure Research Key Laboratory of Infectious Diseases Research in South China, Ministry of Education, Guangdong Provincial Key Laboratory of Viral Hepatitis Research Guangdong Provincial Clinical Research Center for Viral Hepatitis, Guangdong Institute of Hepatology Guangzhou China; 3 School of Nursing and Health Studies Hong Kong Metropolitan University Kowloon Hong Kong China (Hong Kong); 4 Department of Science and Education Shenzhen Baoan Women's and Children's Hospital Shenzhen China

**Keywords:** large language models, LLMs, Chinese National Nursing Licensing Examination, ChatGPT, Qwen-2.5, multiple-choice questions

## Abstract

**Background:**

Large language models (LLMs) have been proposed as valuable tools in medical education and practice. The Chinese National Nursing Licensing Examination (CNNLE) presents unique challenges for LLMs due to its requirement for both deep domain–specific nursing knowledge and the ability to make complex clinical decisions, which differentiates it from more general medical examinations. However, their potential application in the CNNLE remains unexplored.

**Objective:**

This study aims to evaluates the accuracy of 7 LLMs including GPT-3.5, GPT-4.0, GPT-4o, Copilot, ERNIE Bot-3.5, SPARK, and Qwen-2.5 on the CNNLE, focusing on their ability to handle domain-specific nursing knowledge and clinical decision-making. We also explore whether combining their outputs using machine learning techniques can improve their overall accuracy.

**Methods:**

This retrospective cross-sectional study analyzed all 1200 multiple-choice questions from the CNNLE conducted between 2019 and 2023. Seven LLMs were evaluated on these multiple-choice questions, and 9 machine learning models, including Logistic Regression, Support Vector Machine, Multilayer Perceptron, k-nearest neighbors, Random Forest, LightGBM, AdaBoost, XGBoost, and CatBoost, were used to optimize overall performance through ensemble techniques.

**Results:**

Qwen-2.5 achieved the highest overall accuracy of 88.9%, followed by GPT-4o (80.7%), ERNIE Bot-3.5 (78.1%), GPT-4.0 (70.3%), SPARK (65.0%), and GPT-3.5 (49.5%). Qwen-2.5 demonstrated superior accuracy in the Practical Skills section compared with the Professional Practice section across most years. It also performed well in brief clinical case summaries and questions involving shared clinical scenarios. When the outputs of the 7 LLMs were combined using 9 machine learning models, XGBoost yielded the best performance, increasing accuracy to 90.8%. XGBoost also achieved an area under the curve of 0.961, sensitivity of 0.905, specificity of 0.978, *F*_1_-score of 0.901, positive predictive value of 0.901, and negative predictive value of 0.977.

**Conclusions:**

This study is the first to evaluate the performance of 7 LLMs on the CNNLE and that the integration of models via machine learning significantly boosted accuracy, reaching 90.8%. These findings demonstrate the transformative potential of LLMs in revolutionizing health care education and call for further research to refine their capabilities and expand their impact on examination preparation and professional training.

## Introduction

Nursing licensure examinations are essential for maintaining professional standards, ensuring that health care systems are staffed with qualified professionals, and safeguarding patient safety [1]. These examinations assess nurses’ clinical judgment, decision-making, and practical skills, ensuring high-quality care and fostering public trust in the profession [2]. Upholding rigorous standards is critical, as competent health care professionals are crucial for addressing the diverse and complex needs of patients worldwide. The Chinese National Nursing Licensing Examination (CNNLE) plays an important role in maintaining high standards of nursing care in China, ensuring that graduates are well prepared for professional practice [3]. Serving as a benchmark for nursing competence, the CNNLE confirms that nurses possess the necessary skills and knowledge to provide safe and effective care [4]. Beyond its impact on health care quality, the CNNLE also influences educational policies, guiding nursing curricula to meet evolving health care demands. As health care becomes more complex, innovative tools are needed to support the development of skilled professionals capable of providing effective patient care.

The integration of artificial intelligence (AI) in education is transforming learning and assessment, particularly in fields such as nursing [5-7]. ChatGPT, an AI tool that generates content by identifying patterns in its training data, simulates human-like conversations and answers questions across a wide range of topics [8,9]. Its ability to provide correct answers and offer immediate, detailed feedback makes it a valuable resource for students in simulated test environments and question banks [10]. This success in examination settings has sparked interest in using ChatGPT as a self-learning tool, suggesting its potential for enhancing examination preparation and knowledge development [11]. Large language models (LLMs) hold promise for clinical education [12], where these models integrate natural language processing with user-friendly interfaces [13]. In clinics, LLMs are increasingly valuable, particularly in diagnosis [14,15] and clinical licensing examinations [16], where accuracy is crucial. Tools such as ChatGPT are being recognized for their potential to enhance clinical documentation [17], improve diagnostic accuracy [18], and streamline patient care workflows [19]. However, the rapid development of LLMs presents significant challenges in assessing their reliability in the CNNLE.

Passing CNNLE demands not only theoretical knowledge but also clinical decision-making, critical thinking, and practical skills, areas where LLMs often underperform [9]. While tools such as ChatGPT have demonstrated an overall accuracy of 80.75% in nursing education [20], their effectiveness diminishes with complex, context-specific questions requiring nuanced medical knowledge [21-23]. Moreover, concerns regarding patient privacy [24] and biases [25] in LLM outputs raise questions about their suitability for high-stakes assessments such as the CNNLE, which emphasize fairness and accuracy [26,27]. Despite the growing interest in LLMs for medical education, their potential in the CNNLE remains unexplored. Limited understanding exists regarding their ability to handle clinical reasoning, contextual interpretation, and multistep problem-solving in this specific setting. Addressing this gap is crucial to assess their reliability, limitations, and transformative potential in clinical education. Here, this study examines the distribution of question types in the CNNLE from 2019 to 2023 and evaluates the accuracy of 7 LLMs including GPT-3.5, GPT-4.0, GPT-4o, Copilot, ERNIE Bot-3.5, SPARK, and Qwen-2.5—in addressing domain-specific nursing knowledge and clinical decision-making. Furthermore, the study explores whether combining their outputs through machine learning techniques can enhance overall accuracy in this context.

## Methods

### Study Design

This retrospective cross-sectional study evaluated the performance of 7 LLMs on 1200 multiple-choice questions (MCQs) from the CNNLE administered between 2019 and 2023. The study design was chosen for its suitability in systematically analyzing preexisting datasets and providing the capabilities of LLMs across various question types and levels of complexity. A head-to-head evaluation approach was adopted to compare the LLMs. Each MCQ was independently input into each model under identical conditions, ensuring consistency and fairness in the assessment. This parallel evaluation minimized variability caused by external factors, such as differences in question formats or content, allowing for a direct comparison of performance across all models. By using historical data and using a head-to-head evaluation, this study provides an analysis of LLM performance in nursing licensure examinations, offering their potential applications in nursing education and assessment.

### Data Collection

This study analyzed all 1200 MCQs from the CNNLE administered between 2019 and 2023. Each year, 240 MCQs were included, encompassing the 4 question types (A1, A2, A3, and A4) although their proportions varied annually. This comprehensive approach ensured that the evaluation covered diverse question formats and varying levels of complexity, reflecting the full scope of the CNNLE. To ensure the integrity of the evaluation process, 2 researchers (SZ and WH) independently entered each question into 7 LLMs on separate computers. Each question was input into a new chat session to prevent any influence from prior interactions. The LLMs generated answers and explanations solely based on the input questions without pretraining instructions or additional prompts.

If inconsistencies were detected in the responses, a third computer was used to reenter the question in a fresh chat session after clearing the LLMs’ memory. In such cases, the models were instructed to provide more detailed explanations. The researchers then collectively reviewed the answers and explanations to determine the most accurate and contextually appropriate response. When LLMs exhibited confusion, failed to provide explanations, produced multiple answers including the correct one, or encountered specialized queries (eg, questions on local policies), additional instructions were provided. These instructions included prompts such as, “This is a single-choice question. Please select the most suitable or probable answer from options 1 to 5,” “Please choose the incorrect option,” “Tell me the reason why,” “In Chinese local policy,” “In Chinese local law,” and “In Chinese society.” All data generated or analyzed during this study are provided in Multimedia Appendix 1, and the iPython Jupyter notebook code is available in Multimedia Appendix 2.

### Ethical Considerations

The evaluations were conducted between May 15, 2024, and July 17, 2024. All responses were cross-verified against the official CNNLE answer keys. These measures enhanced the reliability and validity of the evaluation process. As this study was purely analytical and did not involve human participants, institutional review board approval and informed consent were not required. All collected data were fully anonymized by removing names, contact details, and other direct identifiers, ensuring no means to reidentify participants.

### Measurements

#### The CNNLE

The CNNLE [28] comprises 2 sections: Professional Practice and Practical Skills, each with 120 questions per unit. The Professional Practice section evaluates a candidate’s ability to implement nursing-related knowledge in clinical settings in a safe and effective manner. It covers medical knowledge related to health and disease, basic nursing skills, and the application of social and humanistic knowledge in nursing practice. The Practical Skills section assesses candidates’ capability to apply nursing knowledge and skills in performing nursing tasks. Topics include clinical manifestations of diseases, treatment principles, health assessment, nursing procedures, professional nursing techniques, and health education. The examination format involves objective questions presented in a computer-based format.

The examination includes 4 question types: A1, A2, A3, and A4, all of which are MCQs. A1 and A2 questions are relatively straightforward, focusing on single knowledge points and brief clinical case summaries, respectively. A3 and A4 questions involve shared clinical scenarios, requiring candidates to analyze and synthesize information comprehensively. A3 questions present 2-3 distinct, patient-centered clinical situations, while A4 questions depict more complex scenarios involving a single patient or family, with 4-6 independent questions that may introduce new information sequentially to test clinical integration skills.

#### LLM Selection

We selected 7 LLMs including GPT-3.5, GPT-4.0, GPT-4o, Copilot, ERNIE Bot-3.5, SPARK, and Qwen-2.5. This diverse selection enabled a comprehensive examination of LLM performance under standardized conditions. GPT-3.5, developed by OpenAI and released in March 2022, is known for generating coherent and contextually relevant text. GPT-4.0, released by OpenAI in March 2023, offers significant improvements in accuracy and understanding. GPT-4o, introduced in May 2024, is an optimized version of GPT-4.0, designed for enhanced performance. ERNIE Bot-3.5, created by Baidu and released in June 2023, is tailored for understanding and generating text in Chinese. SPARK, developed by iFLYTEK and launched in May 2023, enhances performance tools by providing intelligent assistance. Qwen-2.5, created by Alibaba and launched in May 2024, is optimized for complex language understanding, particularly in shopping and customer support contexts. To ensure effectiveness and reliability, each inquiry was conducted only once in a new chat session with each LLM, using 2 different computers. This approach aimed to evaluate the designs’ efficiency in real-world situations without the influence of responses loopholes.

#### Machine Learning Models

We selected 9 machine learning models, each with recognized performance in classification tasks. Logistic Regression (LR) [29] is a fundamental linear model made use of for binary category tasks as a result of its simpleness and interpretability. Support Vector Machine (SVM) [30] excels in high-dimensional and complicated settings, giving durable classification efficiency. Multilayer Perceptron (MLP) [31] is a neural network model that properly identifies complicated patterns with its split structure. The k-nearest neighbors (KNN) [32] algorithm is an uncomplicated, nonparametric monitored knowing approach that categorizes or predicts information factors based upon their proximity to bordering points, extensively acknowledged for its simplicity and effectiveness in both category and regression jobs.

Ensemble models improve prediction performance by incorporating multiple designs to alleviate overfitting and improve generalization. Random Forest (RF) [33] is esteemed for its high precision and ability to alleviate overfitting with ensemble knowing, accumulating the predictions of choice trees using a majority ballot to enhance anticipating toughness. Light Gradient-Boosting Machine (LightGBM) [34] is a highly reliable gradient increasing framework that makes use of a histogram-based technique to bin constant features, speeding up training speed, enhancing memory usage, and mastering processing massive datasets with impressive rate and effectiveness. Adaptive Boosting (AdaBoost) [35] prioritizes tough situations, enhancing category precision by iteratively changing weights to boost the design. Extreme Gradient Boosting (XGBoost) [36], a sophisticated slope-boosting system developed by Chen, iteratively refines designs by splitting tree nodes and suitable residuals, demonstrating extraordinary scalability and superior efficiency throughout varied applications. CatBoost [37], introduced in 2018, is a sophisticated gradient boosting algorithm known for its outstanding handling of specific functions, reduced training times, and the use of a money-grubbing technique to pinpoint ideal tree divides, thereby improving forecast precision.

### Statistical Analysis

The statistical analysis was conducted using Python 3.11.5 (Python Software Foundation) within the Microsoft Visual Studio Code environment. In preparing the dataset, responses where the LLMs failed to provide any answer were categorized as missing values and coded as –1. For valid responses labeled (A, B, C, D, and E), a numerical encoding scheme was applied, converting them to (1, 2, 3, 4, and 5), respectively. To prepare the data for machine learning algorithms, the dataset underwent normalization, scaling all features to a range between 0 and 1 using the MinMaxScaler from the Scikit-learn library. Descriptive statistics were used to analyze the distribution of question types within the CNNLE dataset from 2019 to 2023. Furthermore, accuracy percentages for the LLMs were computed across 2 distinct subjects and 4 different question types. Various machine learning models were then used with the objective of enhancing predictive performance.

Nine machine learning models, including LR, SVM, MLP, KNN, RF, LightGBM, AdaBoost, XGBoost, and CatBoost, were trained specifically for this task using the processed CNNLE dataset. None of the models were pretrained; instead, they were trained and optimized using hyperparameter tuning tailored to the dataset. For instance, parameters such as the number of trees and maximum depth were adjusted for RF, while learning rates and boosting parameters were optimized for LightGBM and XGBoost. The leave-one-out cross-validation method was used to ensure robustness and reliability. The dataset was split into training (90%) and testing (10%) sets, with the training set further divided into 9 subsets for hyperparameter tuning. This iterative process was repeated until each subset served as a validation set, minimizing overfitting and ensuring robust performance metrics for the models.

Model performance was assessed using correlation heatmaps, area under the curves (AUCs), and 7 evaluation metrics: AUC, sensitivity, specificity, *F*_1_-score, accuracy, positive predictive value (PPV), and negative predictive value (NPV). Feature importance was analyzed using Shapley Additive Explanations (SHAP), providing the contributions of individual features. SHAP analysis focused on understanding the relative contributions of the 7 LLMs, highlighting how each LLM’s accuracy influenced overall predictions. The analysis used Python packages, including pandas 2.1.4, numpy 1.24.3, scikit-learn 1.3.0, scipy 1.11.4, catboost 1.2, LightGBM 4.1.0, seaborn 0.12.2, SHAP 0.42.1, and matplotlib 3.8.0.

## Results

### Distribution of Question Types in the CNNLE Over the Years

**Figure 1:**
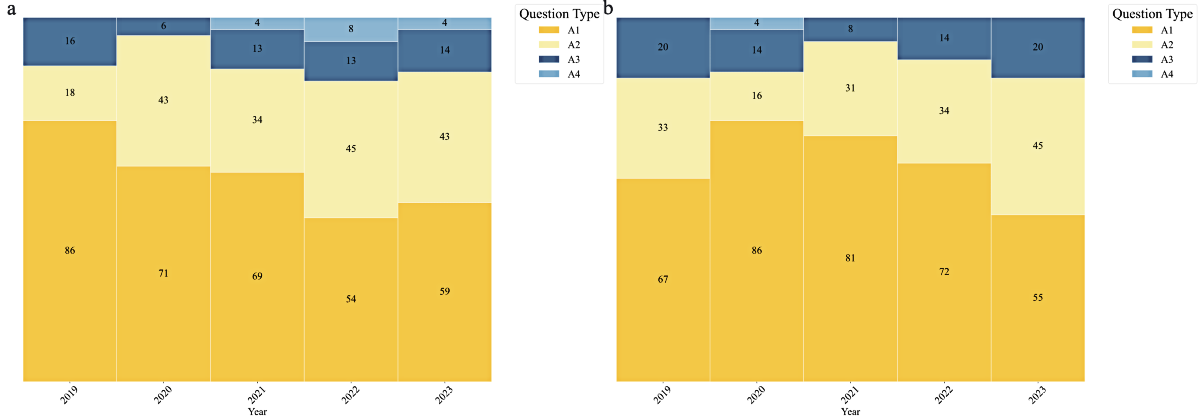
Distribution of question types in CNNLE Professional Practice and Practical Skills sections (2019-2023). (A) Distribution of question types in Practical Skills from 2019 to 2023. (B) Distribution of question types in Professional Practice from 2019 to 2023.

Figure 1 illustrates the distribution of question types over the years in both sections of the CNNLE. Figure 1A depicts the distribution of question types over the years in the Practical Skills section. In the Practical Skills section, A1-type questions decreased from 86 in 2019 to 59 in 2023, while A2-type questions increased from 18 to 43. A3-type and A4-type questions showed smaller fluctuations. Figure 1B shows the distribution of question types over the years in the Professional Practice section. In the Professional Practice section, A1-type questions fell from 67 in 2019 to 55 in 2023, while A2-type questions increased from 33 to 45. A3-type questions remained relatively stable, with minor variations.

### Accuracy of LLMs in Professional Practice

Table 1 presents the accuracy of LLMs in the Professional Practice section from 2019 to 2023. In 2023, Qwen-2.5 achieved the highest accuracy (0.850), followed by ERNIE Bot-3.5 (0.808) and GPT-4o (0.783). GPT-4.0 consistently outperformed GPT-3.5 in all years, with scores of 0.725 and 0.492, respectively, in 2023. Copilot and SPARK also showed moderate performance improvements over time, reaching 0.775 and 0.692 in 2023. Across the 5 years, Qwen-2.5 demonstrated the best overall accuracy (0.875), followed by GPT-4o (0.803) and ERNIE Bot-3.5 (0.785).

**Table 1:** Accuracy of large language models in Professional Practice.

Year	GPT-3.5	GPT-4.0	GPT-4o	Copilot	ERNIE Bot-3.5	SPARK	Qwen-2.5
2023	0.492	0.725	0.783	0.775	0.808	0.692	0.850
2022	0.450	0.675	0.833	0.767	0.758	0.667	0.917
2021	0.517	0.683	0.817	0.725	0.783	0.650	0.900
2020	0.500	0.708	0.725	0.733	0.767	0.600	0.850
2019	0.550	0.758	0.858	0.583	0.808	0.600	0.858
Overall	0.502	0.710	0.803	0.717	0.785	0.642	0.875

### Accuracy of LLMs in Practical Skills

Table 2 presents the accuracy of LLMs in the Practical Skills section from 2019 to 2023. In 2023, Qwen-2.5 achieved the highest accuracy (0.908), followed by GPT-4o (0.833) and Copilot (0.792). GPT-4.0 and ERNIE Bot-3.5 both scored 0.775, showing steady improvement compared with earlier years. SPARK and GPT-3.5 performed moderately, with scores of 0.758 and 0.550, respectively. Over the 5 years, Qwen-2.5 consistently outperformed other models, achieving the highest overall accuracy (0.903). GPT-4o followed with 0.810, while ERNIE Bot-3.5 ranked third with 0.777.

**Table 2:** Accuracy of large language models in Practical Skills.

Year	GPT-3.5	GPT-4.0	GPT-4o	Copilot	ERNIE Bot-3.5	SPARK	Qwen-2.5
2023	0.550	0.775	0.833	0.792	0.775	0.758	0.908
2022	0.467	0.692	0.800	0.792	0.792	0.675	0.850
2021	0.467	0.708	0.850	0.667	0.750	0.567	0.942
2020	0.475	0.642	0.783	0.592	0.800	0.700	0.933
2019	0.483	0.658	0.783	0.458	0.767	0.592	0.883
Overall	0.488	0.695	0.810	0.660	0.777	0.658	0.903

### Accuracy of LLMs for Question Types

Table 3 indicates the accuracy of LLMs across 4 question types (A1, A2, A3, and A4) from 2019 to 2023. In 2023, Qwen-2.5 achieved the highest accuracy for A1, A2, and A3 questions (0.860, 0.909, and 0.853, respectively), while all models reached perfect accuracy (1.000) for A4 questions. GPT-4o consistently performed well across all question types, ranking second or third in accuracy. In 2022, Qwen-2.5 maintained high performance across A1, A2, and A3 questions (0.913, 0.810, and 0.963, respectively). From 2019 to 2021, Qwen-2.5 demonstrated steady improvements across all question types. Overall, Qwen-2.5 achieved the highest average accuracy (0.889), followed by GPT-4o (0.807) and ERNIE Bot-3.5 (0.781).

**Table 3:** Accuracy of large language models for 4 question types.

Question type		GPT-3.5		GPT-4.0	GPT-4o	Copilot	ERNIE Bot-3.5	SPARK	Qwen-2.5
**2023**									
	A1		0.526	0.684	0.789	0.763	0.763	0.719	0.860
	A2		0.443	0.807	0.830	0.784	0.807	0.705	0.909
	A3		0.647	0.794	0.794	0.824	0.824	0.765	0.853
	A4		1.000	1.000	1.000	1.000	1.000	1.000	1.000
**2022**									
	A1		0.476	0.746	0.881	0.825	0.817	0.698	0.913
	A2		0.405	0.582	0.671	0.696	0.671	0.620	0.810
	A3		0.519	0.667	0.889	0.778	0.852	0.630	0.963
	A4		0.500	0.750	1.000	0.875	0.875	0.875	0.875
**2021**									
	A1		0.467	0.660	0.820	0.667	0.727	0.600	0.927
	A2		0.538	0.738	0.846	0.738	0.831	0.646	0.938
	A3		0.524	0.762	0.857	0.714	0.857	0.571	0.810
	A4		0.500	1.000	1.000	1.000	0.750	0.500	1.000
**2020**									
	A1		0.478	0.675	0.771	0.650	0.771	0.656	0.879
	A2		0.492	0.695	0.763	0.695	0.814	0.678	0.915
	A3		0.550	0.600	0.550	0.650	0.750	0.600	0.900
	A4		0.500	0.750	1.000	0.750	1.000	0.250	1.000
**2019**									
	A1		0.490	0.680	0.804	0.503	0.791	0.601	0.869
	A2		0.569	0.745	0.843	0.588	0.784	0.588	0.843
	A3		0.556	0.778	0.861	0.500	0.778	0.583	0.917
Overall		0.495		0.703	0.807	0.688	0.781	0.650	0.889

### Correlation Heatmap and AUC Curves Using Machine Learning

**Figure 2:**
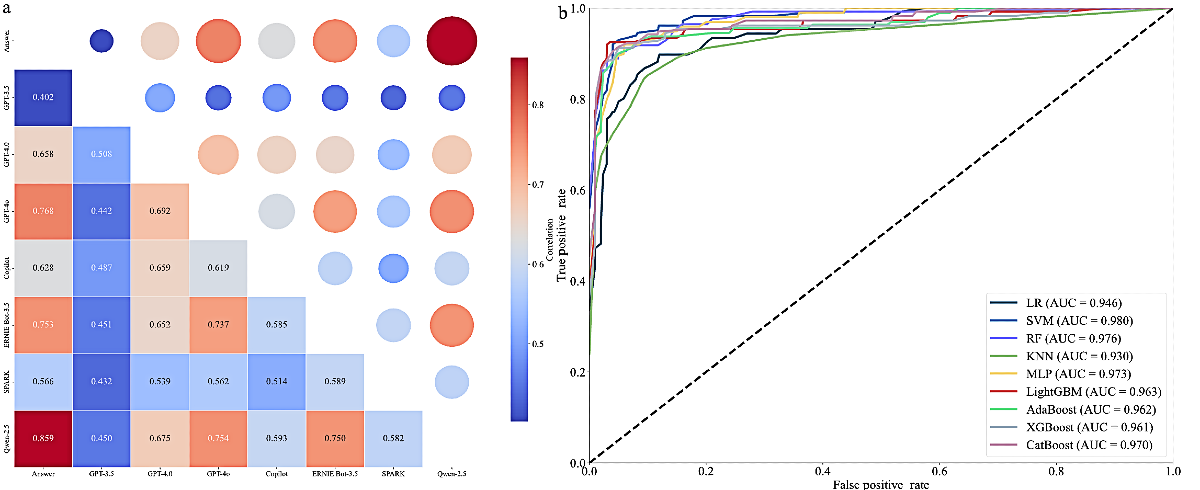
Correlation heatmap and AUC curves of machine learning models in CNNLE. (A) Correlation heatmap: The heatmap illustrates the relationships between different LLMs. The lower left displays numerical correlation values, while the upper right represents correlation magnitude through circle size. Color gradients range from blue (low correlation) to red (high correlation), providing a visual summary of metric interdependencies. (B) AUC curves: The AUC curves compare the performance of various machine learning and ensemble models, highlighting their classification accuracy across the data set. AdaBoost: Adaptive Boosting; AUC: area under the curve; CatBoost: Categorical Boosting; KNN: k-nearest neighbor; LightGBM: Light Gradient-Boosting Machine; LR: Logistic Regression; MLP: Multilayer Perceptron; RF: Random Forest; SVM: Support Vector Machine; XGBoost: Extreme Gradient Boosting.

Figure 2 provides an analysis of the correlation heatmap and AUC curves for machine learning models. Figure 2A presents the correlation heatmap, where Qwen-2.5 shows the highest correlation with correct answers (*r*=0.859), while GPT-3.5 shows the lowest correlation (*r*=0.402). Figure 2B illustrates the AUC scores for each machine learning model in the multiclass classification task. The models achieved the following AUC scores: LR (0.946), SVM (0.980), RF (0.976), KNN (0.930), MLP (0.973), LightGBM (0.963), AdaBoost (0.962), XGBoost (0.961), and CatBoost (0.970).

### Metrics for 5-Class Classification Using Machine Learning

Table 4 presents a comparative analysis of 9 machine learning models for multiclass classification, evaluated by average metrics including AUC, accuracy, sensitivity, specificity, precision, PPV, *F*_1_-score, and NPV. Among these, the SVM and XGBoost models achieve AUC values of 0.980 and 0.961, along with accuracy scores of 0.858 and 0.908, respectively. In contrast, LR and KNN exhibit lower accuracy scores of 0.817 and 0.767.

**Table 4:** Metrics of machine learning.

Classifier	AUC^a^	Accuracy	Sensitivity	Specificity	Precision	PPV^b^	*F*_1_-score	NPV^c^
LR^d^	0.946	0.817	0.808	0.953	0.818	0.818	0.809	0.954
SVM^e^	0.980	0.858	0.857	0.965	0.861	0.861	0.854	0.964
RF^f^	0.976	0.858	0.860	0.965	0.856	0.856	0.854	0.964
KNN^g^	0.930	0.767	0.772	0.942	0.787	0.787	0.768	0.941
MLP^h^	0.973	0.825	0.823	0.957	0.830	0.830	0.819	0.956
LightGBM^i^	0.963	0.900	0.908	0.975	0.895	0.895	0.899	0.974
AdaBoost^j^	0.962	0.858	0.859	0.964	0.855	0.855	0.856	0.964
XGBoost^k^	0.961	0.908	0.905	0.978	0.901	0.901	0.901	0.977
CatBoost^l^	0.970	0.892	0.892	0.974	0.885	0.885	0.885	0.973

^a^AUC: area under the curve.

^b^PPV: positive predictive value.

^c^NPV: negative predictive value.

^d^LR: Logistic Regression.

^e^SVM: Support Vector Machine.

^f^RF: Random Forest.

^g^KNN: k-nearest neighbor.

^h^MLP: Multilayer Perceptron.

^i^LightGBM: Light Gradient-Boosting Machine.

^j^AdaBoost: Adaptive Boosting.

^k^XGBoost: Extreme Gradient Boosting.

^l^CatBoost: Categorical Boosting.

### Importance Ranking of SVM and XGBoost Models

**Figure 3:**
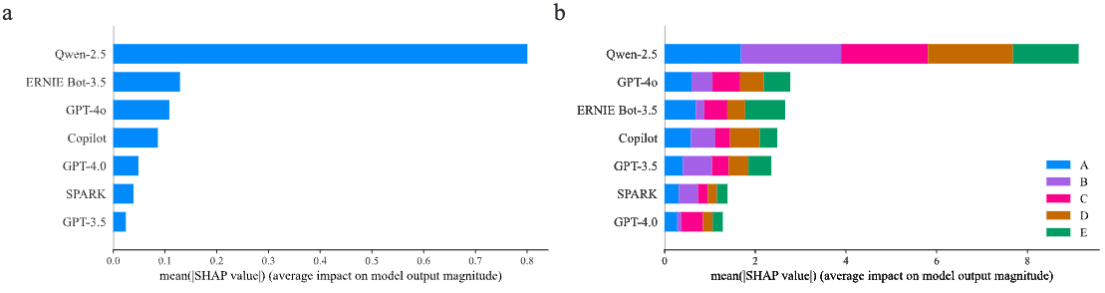
SHAP summary bar plot in Support Vector Machine (SVM) and Extreme Gradient Boosting (XGBoost) models. (A) Importance ranking of SVM model. (B) Importance ranking of XGBoost model. SHAP: Shapley Additive Explanations.

Figure 3 presents the SHAP summary bar plot for both the SVM and XGBoost models. In Figure 3A, the SVM model ranks the features as follows: Qwen-2.5, ERNIE Bot-3.5, GPT-4o, Copilot, GPT-4.0, SPARK, and GPT-3.5. Meanwhile, Figure 3B shows the importance ranking of XGBoost model with a slightly different order: Qwen-2.5, GPT-4o, ERNIE Bot-3.5, Copilot, GPT-3.5, SPARK, and GPT-4.0. Qwen-2.5 stands out as the most influential feature in both models. Furthermore, including other LLMs enhances overall model performance, evidenced by improvements in AUC and accuracy.

## Discussion

### Principal Findings

This study is the first to evaluate the performance of 7 LLMs on the CNNLE dataset (2019-2023), highlighting significant advancements in Chinese LLM development and their applications in nursing education. Among the models tested, Qwen-2.5 demonstrated the highest accuracy (88.92%), significantly surpassing the performance of the other LLMs. This superior accuracy can be attributed to its training on an extensive Chinese dataset and optimized parameters, enabling it to handle domain-specific nursing knowledge and complex clinical decision-making tasks with exceptional precision. These results underline the growing feasibility of deploying advanced LLMs such as Qwen-2.5 to support standardized nursing examinations in China, offering consistent, scalable, and efficient assessments.

Our findings offer a clear pathway for the practical application of Qwen-2.5 in nursing curricula and professional training. For instance, Qwen-2.5 could serve as a virtual tutor, providing personalized feedback and explanations to nursing students in real time. Its ability to respond promptly and accurately to a wide range of questions makes it particularly valuable for addressing individual knowledge gaps and reinforcing complex concepts. Educators could incorporate Qwen-2.5 into classroom activities, using it to simulate clinical scenarios or evaluate students’ decision-making skills. Furthermore, mobile apps powered by Qwen-2.5 could allow nursing students to access high-quality, interactive learning resources anytime and anywhere, thereby enhancing accessibility and flexibility in education. Beyond supporting student learning, Qwen-2.5 and other LLMs can enhance professional development for practicing nurses. For instance, these models could be integrated into continuing education programs, where they act as interactive resources to update practitioners on the latest evidence-based practices. By serving as a knowledge repository, LLMs can enable nurses to quickly access relevant guidelines, ensuring timely and informed clinical decisions.

The results also extend prior research by demonstrating how ensemble machine learning methods can enhance LLM performance in specialized tasks. By integrating the outputs of 7 LLMs using the XGBoost algorithm, we achieved an improved accuracy of 90.83%, surpassing the best-performing single model. This novel application of ensemble methods highlights a promising direction for developing personalized LLMs tailored to specific domains, such as health care education. Previous studies, including those by Li et al [38] and Brin et al [39], have emphasized the value of context-specific tuning, but our research provides concrete evidence of the effectiveness of combining multiple models to enhance accuracy in domain-specific applications.

Furthermore, our study situates Chinese LLMs within the broader global landscape of AI. While skepticism has persisted regarding whether Chinese LLMs can rival models developed by OpenAI or Google, our results demonstrate that Qwen-2.5 not only excels on the CNNLE assessment but also outperforms other LLMs. For example, Qwen-2.5 achieved a higher accuracy than GPT-4 (72.5%) on similar standardized tests, as reported in previous studies [40]. This performance underscores the competitive edge of Chinese-developed models, particularly in addressing language-specific and cultural nuances in health care education.

Our findings also reveal recent trends in nursing education, such as the increasing complexity of standardized examinations such as the CNNLE, which has transitioned from straightforward A1-type questions to more analytical A2-type clinical case scenarios. This shift reflects the growing need for nursing professionals to develop higher-order reasoning skills and apply clinical knowledge to real-world situations. Qwen-2.5’s ability to process nuanced clinical scenarios positions it as an effective tool for addressing these demands. By integrating models such as Qwen-2.5 into nursing curricula, educators can better prepare students for the complexities of modern health care through scenario-based learning and real-time feedback.

Our study also demonstrates the broader impact of China’s advancements in AI, particularly through open access LLMs such as Qwen-2.5 and ERNIE Bot-3.5, which provide practical solutions for addressing regional disparities in nursing education. These models are especially valuable in regions where access to global LLMs, such as OpenAI’s GPT models, is restricted, as they deliver high-quality, localized educational content. By promoting the standardization of nursing education across institutions, these tools help bridge resource gaps and improve the overall quality of health care training in China. Furthermore, their ability to enable personalized and flexible learning through mobile apps empowers students to access educational resources anytime and anywhere. This adaptability positions Chinese LLMs as critical tools for advancing specialized education while addressing both regional inequalities and broader challenges in health care training.

### Limitations

First, our evaluation relied on MCQs to assess the knowledge of LLMs, which may not fully capture their ability to handle open-ended or complex clinical tasks. Future studies could incorporate open-ended questions, clinical simulations, or case-based assessments to evaluate LLMs’ reasoning and decision-making capabilities more comprehensively. These methods would better reflect the unstructured and nuanced scenarios encountered in real-world clinical practice, providing a deeper understanding of how LLMs process complex clinical information. Second, the performance of LLMs can vary based on factors such as prompt design, the number of questions asked, and the context of those questions, introducing variability into results. To address this, standardized evaluation protocols should be developed to ensure consistency across benchmarking studies. Furthermore, future research could focus on refining prompt engineering techniques and optimizing model fine-tuning to improve accuracy and reliability in diverse clinical applications. These refinements could support the development of LLMs that are better suited to handling complex scenarios, such as differential diagnoses or multistep decision-making. Third, while Qwen-2.5 demonstrated highest accuracy on the CNNLE dataset, its optimization for the Chinese language and MCQ format may limit its generalizability to other medical domains and contexts. Future studies should evaluate its applicability in multilingual and open-ended settings to assess its effectiveness in tasks beyond standardized testing formats and within various health care contexts. To enhance the suitability of LLMs for specialized health care tasks, such as diagnostic reasoning and treatment planning, future research could prioritize the development of domain-specific models. This could involve fine-tuning LLMs on datasets that include detailed case histories, diagnostic pathways, and clinical protocols. Such datasets would allow the models to learn context-specific patterns and reasoning processes, equipping them to provide more accurate and relevant recommendations in clinical settings. Furthermore, fine-tuned models could be used to assist in treatment planning by integrating data from clinical guidelines, patient histories, and risk assessment tools to offer tailored suggestions for patient care. Addressing biases in LLM training is also essential for ensuring equitable decision-making across diverse patient populations. Researchers should consider incorporating fairness-aware algorithms and curated datasets that reflect demographic diversity to mitigate potential biases. Such efforts could ensure that domain-specific LLMs provide consistent and unbiased recommendations, particularly in high-stakes environments such as emergency department triage workflows. Finally, this study focused on general purpose LLMs, excluding models explicitly trained for medical tasks, such as Gemini or Claude. Preliminary findings suggest that fine-tuned medical models may achieve superior accuracy for specific applications. Future research should conduct comparative evaluations of general purpose and domain-specific LLMs to identify the optimal approach for different health care needs. These studies could also assess whether fine-tuned models are more effective in real-time clinical workflows, such as triage systems, or in supporting complex decision-making across various medical specialties.

### Conclusions

This study is the first to evaluate the performance of 7 LLMs on the CNNLE and that the integration of models via machine learning significantly boosted accuracy, reaching 90.8%. These findings demonstrate the transformative potential of LLMs in revolutionizing health care education and call for further research to refine their capabilities and expand their impact on examination preparation and professional training.

## Appendix

Multimedia Appendix 1 Original data set.

Multimedia Appendix 2 Ipynb notebook codes.

## Abbreviations

AdaBoost: Adaptive Boosting
AI: artificial intelligence
AUC: area under the curve
CNNLE: Chinese National Nursing Licensing Examination
KNN: k-nearest neighbors
LightGBM: Light Gradient-Boosting Machine
LLM: large language model
LR: Logistic Regression
MCQ: multiple-choice question
MLP: Multilayer Perceptron
NPV: negative predictive value
PPV: positive predictive value
RF: Random Forest
SHAP: Shapley Additive Explanations
SVM: Support Vector Machine
XGBoost: Extreme Gradient Boosting
