# Supplementary material for: Qwen-2.5 Outperforms Other Large Language Models in the Chinese National Nursing Licensing Examination: Retrospective Cross-Sectional Comparative Study
Source: JMIR Med Inform. 2025 Jan 10;13:e63731. doi: 10.2196/63731 (PMC11759905; doi:10.2196/63731)
Supplement: Multimedia Appendix 2 [file medinform_v13i1e63731_app2.pdf]

ipynb codes

August 7, 2024

[27]:
```
import pandas as pd
import matplotlib.pyplot as plt,numpy as np

# Load the dataset
file_path = 'dataset.xlsx'
data = pd.read_excel(file_path)

# Group by 'Year', 'Unit' and count the occurrences of 'Question type'
question_type_counts = data.groupby(['Year', 'Unit', 'Question type']).size().
↪reset_index(name='Count')

# Pivot the data to create a table suitable for a stacked bar plot
pivot_data = question_type_counts.pivot_table(index=['Year', 'Unit'],␣
↪columns='Question type', values='Count', fill_value=0)

# Separate the data by 'Unit'
units = pivot_data.index.get_level_values('Unit').unique()

# Set font properties
plt.rcParams['font.family'] = 'Times New Roman'
plt.rcParams['axes.prop_cycle'] = plt.cycler(color=[
    '#F3C846', '#F7EFAE', '#4A7298', '#8BB5D1'])  # Custom 4 colors
# Initialize counter for saving files
file_counter = 1

# Plot each unit in a separate figure
for unit in units:
    unit_data = pivot_data.xs(unit, level='Unit')
    ax = unit_data.plot(kind='bar', stacked=True, figsize=(20,14), width=1,␣
↪edgecolor='w')

    ax.set_xlabel('Year', fontsize=24)
    ax.set_ylabel('Count', fontsize=24)
    legend = ax.legend(title='Question Type', bbox_to_anchor=(0.99, 1),␣
↪loc='upper left', fontsize=24)  # Adjust legend font size

    # Adjust title font size of the legend
```

```
    plt.setp(legend.get_title(), fontsize=32)

    ax.tick_params(axis='x', rotation=45, labelsize=24)
    ax.tick_params(axis='y', labelsize=24)

    # Remove the top and right spines (outer frames)
    ax.spines['top'].set_visible(False)
    ax.spines['right'].set_visible(False)
    ax.spines['left'].set_visible(False)
    ax.spines['bottom'].set_visible(False)
    ax.yaxis.set_visible(False)  # Hide the y-axis

    # Annotate the bars with counts
    for p in ax.patches:
        width = p.get_width()
        height = p.get_height()
        x, y = p.get_xy()
        if height > 0:  # Only annotate if height is greater than 0
            ax.annotate(f'{height.astype(int)}', (x + width / 2, y + height /␣
↪2), ha='center', va='center', fontsize=24)

    plt.tight_layout()
    # Save the plot for the current unit
    filename = f'fig1{chr(96 + file_counter)}.pdf'  # Generate filenames fig1a.
↪pdf, fig1b.pdf, etc.
    plt.savefig(filename)
    file_counter += 1
plt.show()
```

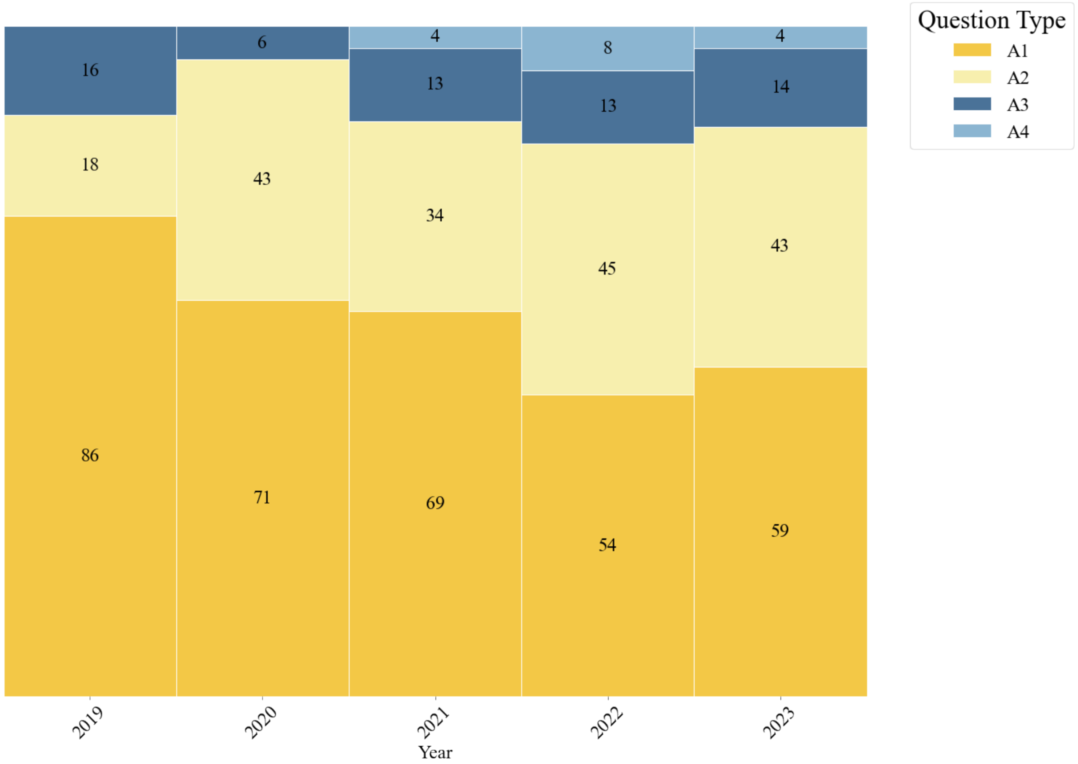
16
6
4
13
8
13
4
14
18
43
34
45
43
86
71
69
54
59
2019
2020
2021
2022
2023
Year
Question Type
A1
A2
A3
A4

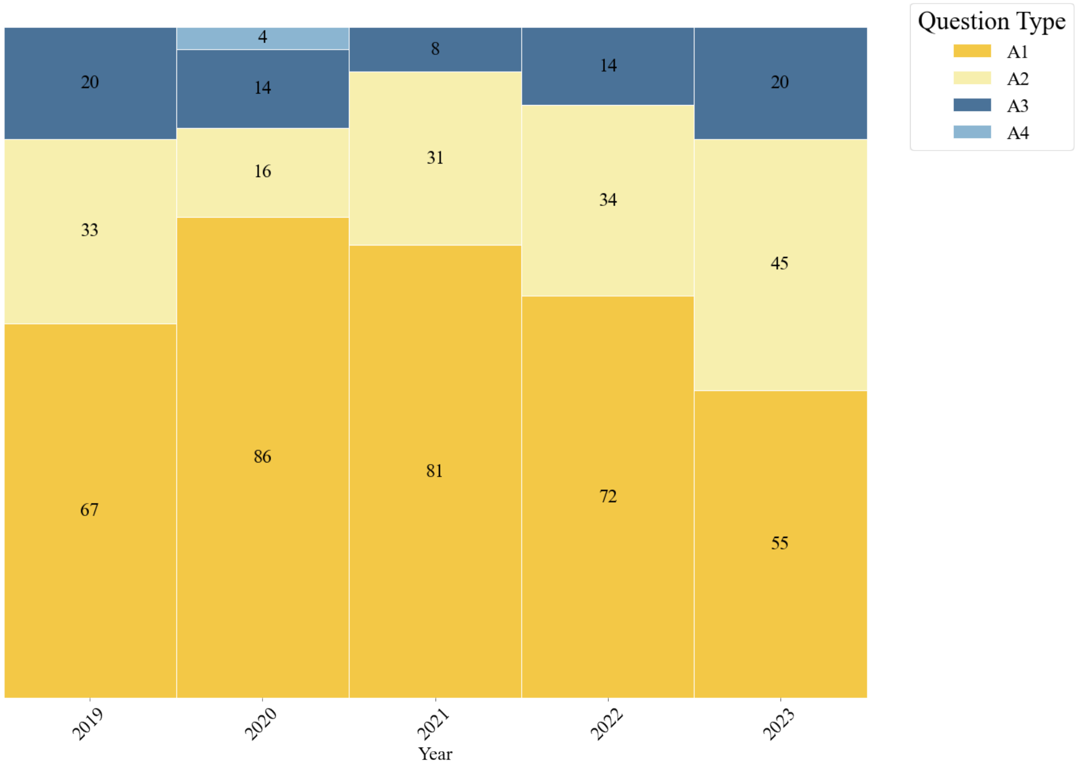
20
4
14
8
14
20
33
16
31
34
45
67
86
81
72
55
2019
2020
2021
2022
2023
Year
Question Type
A1
A2
A3
A4

[6]:

```
import pandas as pd
import matplotlib.pyplot as plt

# Read the Excel file
data = pd.read_excel('dataset.xlsx')

# Replace values that are not A, B, C, D, or E with 'NA'
model_columns = ['GPT 3.5', 'GPT 4.0', 'GPT 4o', 'Copilot', 'ERNIE Bot 3.5',␣
 ↪'SPARK', 'Qwen 2.5']
data[model_columns] = data[model_columns].applymap(lambda x: x if x in ['A',␣
 ↪'B', 'C', 'D', 'E'] else 'NA')

# Calculate accuracy
accuracy_data = {}

for year in data['Year'].unique():
    accuracy_data[year] = {}
    for question_type in data['Unit'].unique():
        filtered_data = data[(data['Year'] == year) & (data['Unit'] ==␣
 ↪question_type)]
        if not filtered_data.empty:
            accuracy_data[year][question_type] = {}
            for model in model_columns:
                correct_answers = (filtered_data[model] ==␣
 ↪filtered_data['Answer']).sum()
                total_answers = len(filtered_data)
                accuracy = correct_answers / total_answers if total_answers > 0␣
 ↪else 0
                accuracy_data[year][question_type][model] = accuracy

# Convert the results to a DataFrame for display
accuracy_df = pd.DataFrame.from_dict({(year, question_type):␣
 ↪accuracy_data[year][question_type]
                                      for year in accuracy_data.keys()
                                      for question_type in accuracy_data[year].
 ↪keys()},
                                     orient='index')

# Get all unique question types
question_types = data['Unit'].unique()

# Define figure names
figure_names = ['fig3a.pdf', 'fig3b.pdf']

# Define colors for the lines
```

```python
colors = ['#8D6278', '#7C7896', '#71919D', '#635C7E', '#628255', '#E2C74B',␣
↪'#AF9392']
markers = ['o', 's', 'D', '^', '*', '+', '>']

# Set font properties globally
plt.rcParams.update({'font.family': 'Times New Roman', 'font.size': 24})

# Plot accuracy over years for each question type without legend
for i, question_type in enumerate(question_types):
    plt.figure(figsize=(20, 12))
    for j, model in enumerate(model_columns):
        years = sorted(accuracy_df.loc[accuracy_df.index.get_level_values(1) ==␣
↪question_type].index.get_level_values(0))
        accuracies = [accuracy_df.loc[(year, question_type)][model] for year in␣
↪years]
        plt.plot(years, accuracies, marker=markers[j], markersize=18,␣
↪markeredgewidth=4, linestyle='-', linewidth=4, label=model, color=colors[j])

    plt.axhline(y=0.6, color='black', linestyle='--', linewidth=4)  # Add␣
↪horizontal dashed line at y=0.6
    plt.axhline(y=0.8, color='green', linestyle='--', linewidth=4)  # Add␣
↪horizontal dashed line at y=0.8
    plt.xlabel('Year', fontsize=24)
    plt.ylabel('Accuracy', fontsize=24)
    print(f'Accuracy Over Years for Unit {question_type}')
    plt.xticks(fontsize=24)
    plt.yticks(fontsize=24)
    plt.grid(False)
    plt.gca().xaxis.set_major_locator(plt.MaxNLocator(integer=True))  # Ensure␣
↪x-axis shows only integers
    #plt.savefig(figure_names[i], bbox_inches='tight')
    plt.close()

# Create a separate legend figure
plt.figure(figsize=(20,12))
for j, model in enumerate(model_columns):
    plt.plot([], [], label=model, color=colors[j], marker=markers[j],␣
↪markersize=24, markeredgewidth=4, linestyle='-', linewidth=4)
plt.legend(loc='center', fontsize=24)
plt.axis('off')
#plt.savefig('fig3legend1.pdf', bbox_inches='tight')
plt.close()
display(accuracy_df)
```

```
Accuracy Over Years for Unit Professional Practice
Accuracy Over Years for Unit Practical Skills
                         GPT 3.5   GPT 4.0    GPT 4o   Copilot   \
```

```
2023 Professional Practice  0.491667  0.725000  0.783333  0.775000
     Practical Skills       0.550000  0.775000  0.833333  0.791667
2022 Professional Practice  0.450000  0.675000  0.833333  0.766667
     Practical Skills       0.466667  0.691667  0.800000  0.791667
2021 Professional Practice  0.516667  0.683333  0.816667  0.725000
     Practical Skills       0.466667  0.708333  0.850000  0.666667
2020 Professional Practice  0.500000  0.708333  0.725000  0.733333
     Practical Skills       0.475000  0.641667  0.783333  0.591667
2019 Professional Practice  0.550000  0.758333  0.858333  0.583333
     Practical Skills       0.483333  0.658333  0.783333  0.458333

                            ERNIE Bot 3.5     SPARK  Qwen 2.5
2023 Professional Practice       0.808333  0.691667  0.850000
     Practical Skills            0.775000  0.758333  0.908333
2022 Professional Practice       0.758333  0.666667  0.916667
     Practical Skills            0.791667  0.675000  0.850000
2021 Professional Practice       0.783333  0.650000  0.900000
     Practical Skills            0.750000  0.566667  0.941667
2020 Professional Practice       0.766667  0.600000  0.850000
     Practical Skills            0.800000  0.700000  0.933333
2019 Professional Practice       0.808333  0.600000  0.858333
     Practical Skills            0.766667  0.591667  0.883333
```

[8]:

```python
import pandas as pd
import matplotlib.pyplot as plt

# Read the Excel file
data = pd.read_excel('dataset.xlsx')

# Replace values that are not A, B, C, D, or E with 'NA'
model_columns = ['GPT 3.5', 'GPT 4.0', 'GPT 4o', 'Copilot', 'ERNIE Bot 3.5',␣
↪'SPARK', 'Qwen 2.5']
data[model_columns] = data[model_columns].applymap(lambda x: x if x in ['A',␣
↪'B', 'C', 'D', 'E'] else 'NA')

# Calculate accuracy
accuracy_data = {}

for year in data['Year'].unique():
    accuracy_data[year] = {}
    for question_type in data['Question type'].unique():
        filtered_data = data[(data['Year'] == year) & (data['Question type'] ==␣
↪question_type)]
        if not filtered_data.empty:
            accuracy_data[year][question_type] = {}
            for model in model_columns:
```

```
                correct_answers = (filtered_data[model] ==␣
↪filtered_data['Answer']).sum()
                total_answers = len(filtered_data)
                accuracy = correct_answers / total_answers if total_answers > 0␣
↪else 0
                accuracy_data[year][question_type][model] = accuracy

# Convert the results to a DataFrame for display
accuracy_df = pd.DataFrame.from_dict({(year, question_type):␣
↪accuracy_data[year][question_type]
                                     for year in accuracy_data.keys()
                                     for question_type in accuracy_data[year].
↪keys()},
                                    orient='index')

# Get all unique question types
question_types = data['Question type'].unique()

# Define figure names
figure_names = ['fig3c.pdf', 'fig3d.pdf', 'fig3e.pdf', 'fig3f.pdf']

# Define colors for the lines
colors = ['#8D6278', '#7C7896', '#71919D', '#635C7E', '#628255', '#E2C74B',␣
↪'#AF9392']
markers = ['o', 's', 'D', '^', '*', '+', '>']
# Set font properties globally
plt.rcParams.update({'font.family': 'Times New Roman', 'font.size': 24})
# Plot accuracy over years for each question type without legend
for i, question_type in enumerate(question_types):
    plt.figure(figsize=(20, 12))
    for j, model in enumerate(model_columns):
        years = sorted(accuracy_df.loc[accuracy_df.index.get_level_values(1) ==␣
↪question_type].index.get_level_values(0))
        accuracies = [accuracy_df.loc[(year, question_type)][model] for year in␣
↪years]
        plt.plot(years, accuracies, marker=markers[j], markersize=18,␣
↪markeredgewidth=4, linestyle='-', linewidth=4, label=model, color=colors[j])

    plt.axhline(y=0.6, color='black', linestyle='--', linewidth=4)  # Add␣
↪horizontal dashed line at y=0.6
    plt.axhline(y=0.8, color='green', linestyle='--', linewidth=4)  # Add␣
↪horizontal dashed line at y=0.8
    plt.xlabel('Year', fontsize=24)
    plt.ylabel('Accuracy', fontsize=24)
    print(f'Accuracy Over Years for Question Type {question_type}')
    plt.xticks(fontsize=24)
```

```python
    plt.yticks(fontsize=24)
    plt.grid(False)
    plt.gca().xaxis.set_major_locator(plt.MaxNLocator(integer=True))  # Ensure x-axis shows only integers
    #plt.savefig(figure_names[i], bbox_inches='tight')
    plt.close()

# Create a separate legend figure
plt.figure(figsize=(20,12))
for j, model in enumerate(model_columns):
    plt.plot([], [], label=model, color=colors[j], marker=markers[j], markersize=24, markeredgewidth=4, linestyle='-', linewidth=4)
plt.legend(loc='center', fontsize=24)
plt.axis('off')
#plt.savefig('fig3legend2.pdf', bbox_inches='tight')
plt.close()
display(accuracy_df)
#accuracy_df.to_csv('accuracy.csv')
```

```
Accuracy Over Years for Question Type A1
Accuracy Over Years for Question Type A2
Accuracy Over Years for Question Type A3
Accuracy Over Years for Question Type A4
```

```
          GPT 3.5   GPT 4.0    GPT 4o   Copilot  ERNIE Bot 3.5     SPARK  \
2023 A1  0.526316  0.684211  0.789474  0.763158       0.763158  0.719298
     A2  0.443182  0.806818  0.829545  0.784091       0.806818  0.704545
     A3  0.647059  0.794118  0.794118  0.823529       0.823529  0.764706
     A4  1.000000  1.000000  1.000000  1.000000       1.000000  1.000000
2022 A1  0.476190  0.746032  0.880952  0.825397       0.817460  0.698413
     A2  0.405063  0.582278  0.670886  0.696203       0.670886  0.620253
     A3  0.518519  0.666667  0.888889  0.777778       0.851852  0.629630
     A4  0.500000  0.750000  1.000000  0.875000       0.875000  0.875000
2021 A1  0.466667  0.660000  0.820000  0.666667       0.726667  0.600000
     A2  0.538462  0.738462  0.846154  0.738462       0.830769  0.646154
     A3  0.523810  0.761905  0.857143  0.714286       0.857143  0.571429
     A4  0.500000  1.000000  1.000000  1.000000       0.750000  0.500000
2020 A1  0.477707  0.675159  0.770701  0.649682       0.770701  0.656051
     A2  0.491525  0.694915  0.762712  0.694915       0.813559  0.677966
     A3  0.550000  0.600000  0.550000  0.650000       0.750000  0.600000
     A4  0.500000  0.750000  1.000000  0.750000       1.000000  0.250000
2019 A1  0.490196  0.679739  0.803922  0.503268       0.790850  0.601307
     A2  0.568627  0.745098  0.843137  0.588235       0.784314  0.588235
     A3  0.555556  0.777778  0.861111  0.500000       0.777778  0.583333

         Qwen 2.5
2023 A1  0.859649
     A2  0.909091
```

```
     A3   0.852941
     A4   1.000000
2022 A1   0.912698
     A2   0.810127
     A3   0.962963
     A4   0.875000
2021 A1   0.926667
     A2   0.938462
     A3   0.809524
     A4   1.000000
2020 A1   0.878981
     A2   0.915254
     A3   0.900000
     A4   1.000000
2019 A1   0.869281
     A2   0.843137
     A3   0.916667
```

[12]:

```
import pandas as pd
import matplotlib.pyplot as plt
import numpy as np
import seaborn as sns
from scipy.stats import kruskal as k_w_test

# Read the Excel file
df = pd.read_excel('dataset.xlsx')

# Rename columns
cols_to_check = ['GPT-3.5', 'GPT-4.0', 'GPT-4o', 'Copilot', 'ERNIE Bot-3.5',␣
↪'SPARK', 'Qwen-2.5']
df.rename(columns={'GPT 3.5': 'GPT-3.5', 'GPT 4.0': 'GPT-4.0', 'GPT 4o':␣
↪'GPT-4o',
                   'ERNIE Bot 3.5': 'ERNIE Bot-3.5', 'Qwen 2.5': 'Qwen-2.5'},␣
↪inplace=True)

# Replace values not in A, B, C, D, E with NA
df[cols_to_check] = df[cols_to_check].applymap(lambda x: x if x in ['A', 'B',␣
↪'C', 'D', 'E'] else 'NA')

# Set font properties globally
plt.rcParams.update({'font.family': 'Times New Roman', 'font.size': 24})

# Calculate accuracy for each model
accuracy_results = {}
for col in cols_to_check:
    correct = (df[col] == df['Answer']).sum()
    total = len(df)
```

```
    accuracy = correct / total if total > 0 else 0
    accuracy_results[col] = accuracy

# Convert the accuracy results to a DataFrame for display
accuracy_df = pd.DataFrame(list(accuracy_results.items()), columns=['Model',␣
↪'Accuracy'])

# Display the accuracy results
print("Accuracy Results:")
display(accuracy_df)

# Plot the results using a bar plot
fig, ax = plt.subplots(figsize=(12, 8))

# Define colors for the bars
colors = ['#8D6278', '#7C7896', '#71919D', '#635C7E', '#628255', '#E2C74B',␣
↪'#AF9392']

bars = ax.bar(accuracy_df['Model'], accuracy_df['Accuracy'], color=colors)
ax.set_xlabel('LLMs')
ax.set_ylabel('Overall Accuracy')

plt.xticks(rotation=45)
#plt.savefig('fig2.pdf', bbox_inches='tight')
plt.close()

# Encode 'A', 'B', 'C', 'D', 'E' as 1, 2, 3, 4, 5 and 'NA' as 0
encoding = {'A': 1, 'B': 2, 'C': 3, 'D': 4, 'E': 5, 'NA': 0}
df[cols_to_check] = df[cols_to_check].applymap(lambda x: encoding[x])

# Perform Kruskal-Wallis test between all pairs of models
k_w_test_results = {}
for i in range(len(cols_to_check)):
    for j in range(i+1, len(cols_to_check)):
        model1 = cols_to_check[i]
        model2 = cols_to_check[j]
        stat, p_value = k_w_test(df[model1], df[model2])
        k_w_test_results[(model1, model2)] = {'stat': stat, 'p_value': p_value}

# Save the K-W test results to a DataFrame and then to an Excel file
k_w_test_df = pd.DataFrame.from_dict(k_w_test_results, orient='index')
display(k_w_test_df)
```

```
Accuracy Results:

           Model  Accuracy
0        GPT-3.5  0.495000
1        GPT-4.0  0.702500
```

```
2          GPT-4o  0.806667
3         Copilot  0.688333
4   ERNIE Bot-3.5  0.780833
5           SPARK  0.650000
6        Qwen-2.5  0.889167

                                stat   p_value
GPT-3.5       GPT-4.0        0.271881  0.602073
              GPT-4o         1.765290  0.183966
              Copilot        0.270228  0.603179
              ERNIE Bot-3.5  0.156220  0.692661
              SPARK          0.270901  0.602728
              Qwen-2.5       0.493968  0.482163
GPT-4.0       GPT-4o         3.495367  0.061541
              Copilot        1.091561  0.296126
              ERNIE Bot-3.5  0.834323  0.361025
              SPARK          1.140985  0.285444
              Qwen-2.5       1.566054  0.210781
GPT-4o        Copilot        0.673634  0.411787
              ERNIE Bot-3.5  0.867499  0.351648
              SPARK          0.686271  0.407436
              Qwen-2.5       0.422181  0.515851
Copilot       ERNIE Bot-3.5  0.015687  0.900326
              SPARK          0.000243  0.987559
              Qwen-2.5       0.039942  0.841595
ERNIE Bot-3.5 SPARK          0.018312  0.892358
              Qwen-2.5       0.103687  0.747449
SPARK         Qwen-2.5       0.028374  0.866233
```

[30]:

```python
import pandas as pd
import numpy as np
import matplotlib.pyplot as plt
import seaborn as sns
from scipy.stats import ttest_rel

# Read the Excel file
data = pd.read_excel('dataset.xlsx')

# Replace values that are not A, B, C, D, or E with 'NA'
model_columns = ['Answer', 'GPT 3.5', 'GPT 4.0', 'GPT 4o', 'Copilot', 'ERNIE␣
↪Bot 3.5', 'SPARK', 'Qwen 2.5']
data = data[model_columns].applymap(lambda x: x if x in ['A', 'B', 'C', 'D',␣
↪'E'] else 'NA')
data.rename(columns={'GPT 3.5': 'GPT-3.5', 'GPT 4.0': 'GPT-4.0', 'GPT 4o':␣
↪'GPT-4o',
                    'ERNIE Bot 3.5': 'ERNIE Bot-3.5', 'Qwen 2.5': 'Qwen-2.5'},␣
↪inplace=True)
```

```
# Update model_columns after renaming
model_columns = ['Answer', 'GPT-3.5', 'GPT-4.0', 'GPT-4o', 'Copilot', 'ERNIE␣
↪Bot-3.5', 'SPARK', 'Qwen-2.5']

# Encode 'A', 'B', 'C', 'D', 'E' as 1, 2, 3, 4, 5 and 'NA' as 0
encoding = {'A': 1, 'B': 2, 'C': 3, 'D': 4, 'E': 5, 'NA': 0}
data[model_columns] = data[model_columns].applymap(lambda x: encoding[x])

# Calculate the correlation matrix
corr_matrix = data.corr()

# Create a mask for the upper triangle
mask = np.triu(np.ones_like(corr_matrix, dtype=bool))

# Set up the matplotlib figure
plt.figure(figsize=(20, 16))

# Set font properties globally
plt.rcParams.update({'font.family': 'Times New Roman', 'font.size': 18})

# Draw the heatmap with the mask and correct aspect ratio
heatmap = sns.heatmap(corr_matrix, mask=mask, annot=True, fmt='.2f',␣
↪cmap='coolwarm',
                      cbar_kws={"shrink": .8}, linewidths=.5)

# Customize x and y axis label fonts
heatmap.set_xticklabels(heatmap.get_xticklabels(), fontsize=14)
heatmap.set_yticklabels(heatmap.get_yticklabels(), fontsize=14)

# Customize the legend font
colorbar = heatmap.collections[0].colorbar
colorbar.ax.tick_params(labelsize=18)
colorbar.ax.yaxis.set_tick_params(labelsize=18)
colorbar.set_label('Correlation', fontsize=18)

# Add circles proportional to the correlation values with filled color
for i in range(len(corr_matrix.columns)):
    for j in range(i+1, len(corr_matrix.columns)):
        corr_value = corr_matrix.iloc[i, j]
        color = heatmap.collections[0].cmap(heatmap.collections[0].
↪norm(corr_value))
        plt.gca().add_patch(plt.Circle((j+0.5, i+0.5), radius=abs(corr_value) *␣
↪0.5, color=color, ec=color))

plt.savefig('fig2a.pdf', bbox_inches='tight')
plt.show()
```

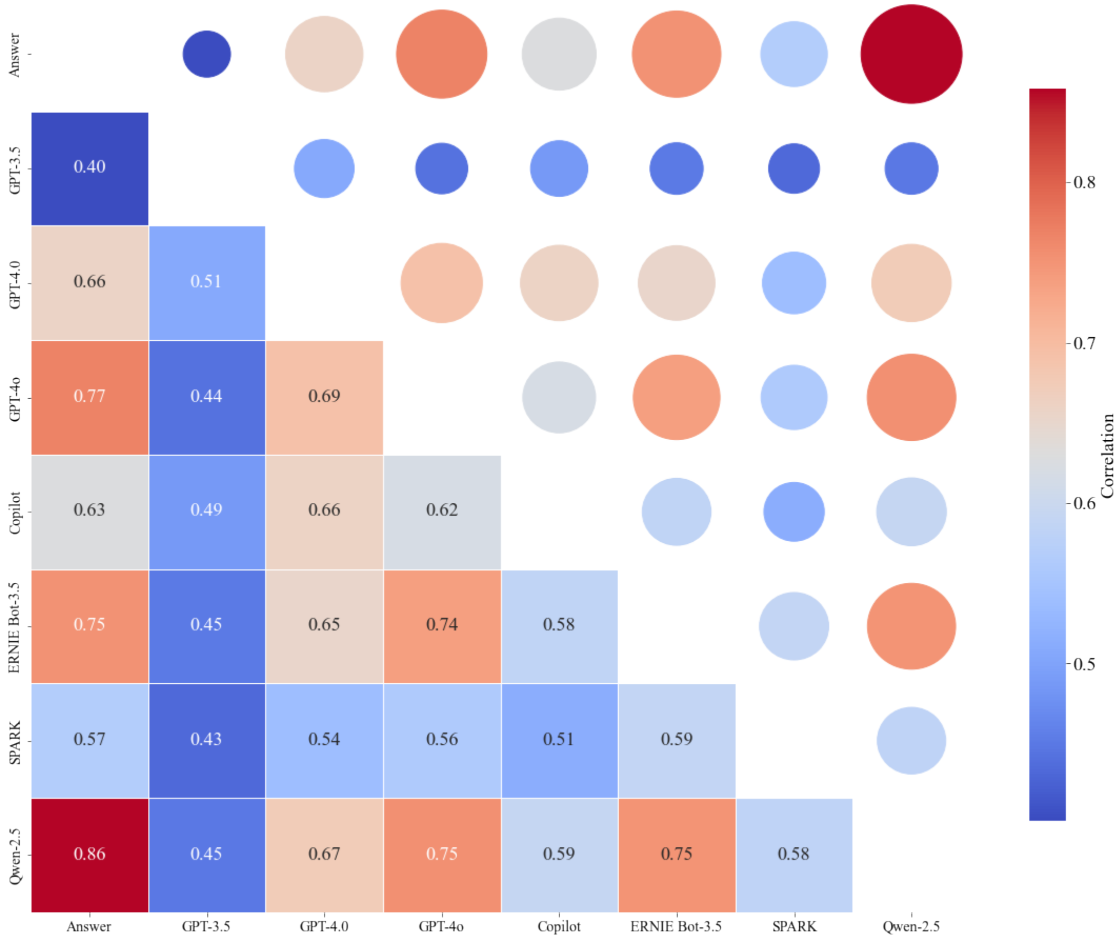

[35]:

```
import warnings
warnings.filterwarnings('ignore')
import pandas as pd
import numpy as np
import matplotlib.pyplot as plt
from sklearn.model_selection import StratifiedKFold
from sklearn.preprocessing import label_binarize, LabelEncoder, MinMaxScaler
from sklearn.metrics import (
    accuracy_score, f1_score, confusion_matrix,
    roc_curve, auc
)
from sklearn.ensemble import RandomForestClassifier, AdaBoostClassifier
from sklearn.linear_model import LogisticRegression
from sklearn.svm import SVC
from sklearn.neighbors import KNeighborsClassifier
from sklearn.neural_network import MLPClassifier
from lightgbm import LGBMClassifier
```

```python
from xgboost import XGBClassifier
from catboost import CatBoostClassifier

# Read the Excel file
data = pd.read_excel('dataset.xlsx')

# Replace values that are not A, B, C, D, or E with 'NA'
model_columns = ['Answer', 'GPT 3.5', 'GPT 4.0', 'GPT 4o', 'Copilot', 'ERNIE Bot 3.5', 'SPARK', 'Qwen 2.5']
data = data[model_columns].applymap(lambda x: x if x in ['A', 'B', 'C', 'D', 'E'] else 'NA')

# Encode 'A', 'B', 'C', 'D', 'E' as 1, 2, 3, 4, 5 and 'NA' as 0
encoding = {'A': 1, 'B': 2, 'C': 3, 'D': 4, 'E': 5, 'NA': 0}
data[model_columns] = data[model_columns].applymap(lambda x: encoding[x])

# Apply MinMaxScaler scaling
minmax_scaler = MinMaxScaler()

# Prepare the data for modeling
X = data.drop(columns=['Answer'])
X = minmax_scaler.fit_transform(X)

y = LabelEncoder().fit_transform(data['Answer'])

# Define a dictionary of classifiers
classifiers = {
    "LR": LogisticRegression(),
    "SVM": SVC(probability=True),
    "RF": RandomForestClassifier(),
    'KNN': KNeighborsClassifier(),
    "MLP": MLPClassifier(),
    "LightGBM": LGBMClassifier(),
    "AdaBoost": AdaBoostClassifier(),
    "XGBoost": XGBClassifier(),
    "CatBoost": CatBoostClassifier(silent=True)
}

colors = ["#14517C", "#2F7FC1", "#8E8BFE", "#96C37D", "#F3D266", "#D8383A", "#63E398", "#A9B8C6", "#C497B2"]

results = []  # List to store evaluation results

# Prepare to plot ROC curves
plt.figure(figsize=(20,16))
plt.rcParams.update({'font.family': 'Times New Roman', 'font.size': 24})
```

```
# Perform 10-fold cross-validation
skf = StratifiedKFold(n_splits=10)

for i, (clf_name, clf) in enumerate(classifiers.items()):  # Use enumerate to
↪keep track of the index
    aucs = []
    accuracies = []
    sensitivities = []
    specificities = []
    precisions = []
    f1_scores = []
    npvs = []
    ppvs = []  # To store PPV (Precision) values

    for train_index, test_index in skf.split(X, y):
        X_train, X_test = X[train_index], X[test_index]
        y_train, y_test = y[train_index], y[test_index]

        clf.fit(X_train, y_train)
        y_pred_proba = clf.predict_proba(X_test)
        y_pred = clf.predict(X_test)

        n_classes = len(np.unique(y))
        y_test_bin = label_binarize(y_test, classes=range(n_classes))

        # Compute ROC curve and area under the curve for each class
        fpr = dict()
        tpr = dict()
        roc_auc = dict()
        for j in range(n_classes):
            fpr[j], tpr[j], _ = roc_curve(y_test_bin[:, j], y_pred_proba[:, j])
            roc_auc[j] = auc(fpr[j], tpr[j])

        # Compute average ROC AUC
        all_fpr = np.unique(np.concatenate([fpr[j] for j in range(n_classes)]))
        mean_tpr = np.zeros_like(all_fpr)
        for j in range(n_classes):
            mean_tpr += np.interp(all_fpr, fpr[j], tpr[j])
        mean_tpr /= n_classes
        aucs.append(auc(all_fpr, mean_tpr))

        # Compute metrics using one-vs-rest approach
        cm = confusion_matrix(y_test, y_pred)
        specificity_list = []
        npv_list = []
        precision_list = []
        recall_list = []
```

```
        f1_list = []
        ppv_list = []

        for j in range(n_classes):
            # One-vs-rest approach
            tp = cm[j, j]
            fp = cm[:, j].sum() - tp
            fn = cm[j, :].sum() - tp
            tn = cm.sum() - tp - fp - fn

            specificity = tn / (tn + fp) if (tn + fp) != 0 else 0
            npv = tn / (tn + fn) if (tn + fn) != 0 else 0
            ppv = tp / (tp + fp) if (tp + fp) != 0 else 0
            precision = tp / (tp + fp) if (tp + fp) != 0 else 0
            recall = tp / (tp + fn) if (tp + fn) != 0 else 0
            f1 = 2 * (precision * recall) / (precision + recall) if (precision␣
↪+ recall) != 0 else 0

            specificity_list.append(specificity)
            npv_list.append(npv)
            precision_list.append(precision)
            recall_list.append(recall)
            f1_list.append(f1)
            ppv_list.append(ppv)

        # Compute average metrics across all classes
        specificity_avg = np.mean(specificity_list)
        npv_avg = np.mean(npv_list)
        precision_avg = np.mean(precision_list)
        recall_avg = np.mean(recall_list)
        f1_avg = np.mean(f1_list)
        ppv_avg = np.mean(ppv_list)

        accuracies.append(accuracy_score(y_test, y_pred))
        sensitivities.append(recall_avg)
        specificities.append(specificity_avg)
        precisions.append(precision_avg)
        f1_scores.append(f1_avg)
        npvs.append(npv_avg)
        ppvs.append(ppv_avg)

    # Store the average results for this classifier
    results.append({
        "Classifier": clf_name,
        "AUC": np.mean(aucs),
        "Accuracy": np.mean(accuracies),
        "Sensitivity (Recall)": np.mean(sensitivities),
```

```
        "Specificity": np.mean(specificities),
        "Precision": np.mean(precisions),
        "PPV":np.mean(ppvs),
        "F1 Score": np.mean(f1_scores),
        "NPV": np.mean(npvs)
    })

    # Plot ROC curve for each classifier with unique color
    plt.plot(all_fpr, mean_tpr, label=f'{clf_name} (AUC = {np.mean(aucs):.
 ↪3f})', linewidth=4, color=colors[i % len(colors)])

# Finalize ROC plot
plt.plot([0, 1], [0, 1], 'k--', linewidth=4)  # Dashed diagonal
plt.xlabel('False Positive Rate')
plt.ylabel('True Positive Rate')
plt.xlim(0, 1)
plt.ylim(0, 1)
plt.legend(loc="lower right")
plt.savefig('fig2b.pdf', bbox_inches='tight')
plt.show()

# Display the results
results_df = pd.DataFrame(results)
print('Metric Results:')
display(results_df)
```

[106]:

```
import shap
import matplotlib.pyplot as plt

# SHAP analysis for CatBoost model
catboost_model = classifiers["CatBoost"]
explainer = shap.TreeExplainer(catboost_model)
shap_values = explainer.shap_values(X_test)

# Summary plot for each class (bar plot) with different colors
class_names = ['A', 'B', 'C', 'D', 'E']
colors = ['#2878B5','#9AC9DB','#F8AC8C','#C82423','#FF8884']

# Create a summary plot and save it
shap.summary_plot(shap_values, X_test, plot_type='bar',class_names=class_names,␣
 ↪feature_names=['GPT 3.5', 'GPT 4.0', 'GPT 4o', 'Copilot', 'ERNIE Bot 3.5',␣
 ↪'SPARK', 'Qwen 2.5'],show=False)
plt.savefig('fig6d.pdf')
plt.show()
```

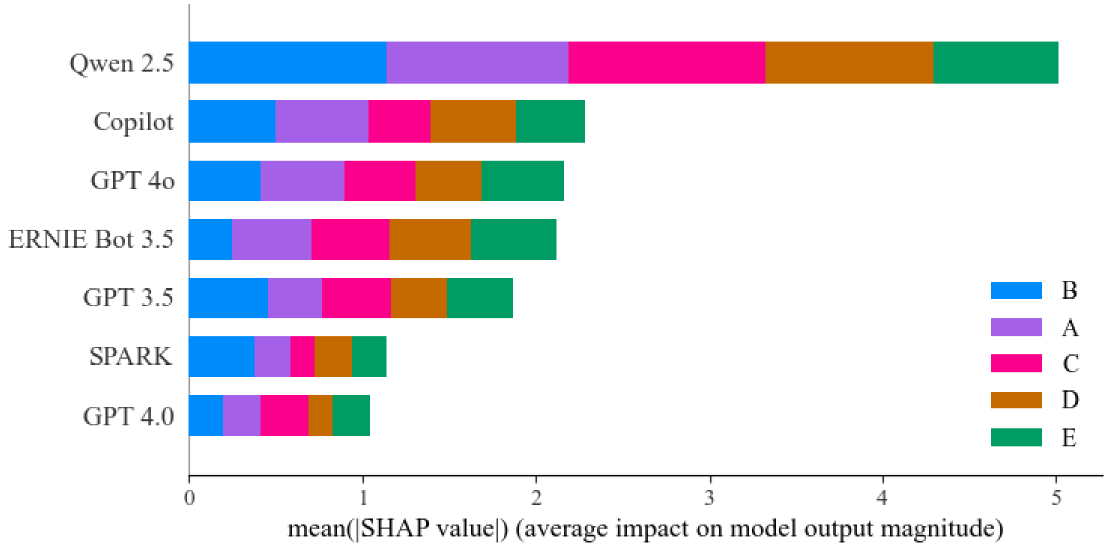
Qwen 2.5
Copilot
GPT 4o
ERNIE Bot 3.5
GPT 3.5
SPARK
GPT 4.0
B
A
C
D
E
0
1
2
3
4
5
mean(|SHAP value|) (average impact on model output magnitude)
